# Mechanical massage and mental training programmes affect employees’ anxiety, stress susceptibility and detachment–a randomised explorative pilot study

**DOI:** 10.1186/s12906-015-0753-x

**Published:** 2015-09-02

**Authors:** Jasmin Muller, Linda Handlin, Mikael Harlén, Ulrika Lindmark, Anette Ekström

**Affiliations:** School of Health and Education, University of Skövde, S-541 28 Skövde, Sweden; School of Health Sciences, Jönköping University, S-551 11 Jönköping, Sweden

**Keywords:** Stress, Anxiety, Alternative, Complementary, Intervention, Physical Health, Psychosocial health, Working place

## Abstract

**Background:**

Working people’s reduced ability to recover has been proposed as a key factor behind the increase in stress-related health problems. One not yet evidence-based preventive method designed to help employees keep healthy and be less stressed is an armchair with built-in mechanical massage and mental training programmes, This study aimed to evaluate possible effects on employees’ experience of levels of “Anxiety”, “Stress Susceptibility”, “Detachment” and “Social Desirability” when using mechanical massage and mental training programmes, both separately and in combination, during working hours.

**Methods:**

Employees from four different workplaces were randomly assigned to one of the following groups: *i) Massage and mental training* (sitting in the armchair and receiving mechanical massage while listening to the mental training programmes, *n* = 19), *ii*) *Massage* (sitting in the armchair and receiving mechanical massage only, *n* = 19), *iii*) Mental training (sitting in the armchair and listening to the mental training programmes only, *n* = 19), *iv*) *Pause* (sitting in the armchair but not receiving mechanical massage or listening to the mental training programmes, *n* = 19), *v*) *Control* (not sitting in the armchair at all, *n* = 17). In order to discover how the employees felt about their own health they were asked to respond to statements from the ”Swedish Scale of Personality” (SSP), immediately before the randomisation, after four weeks and after eight weeks (end-of-study).

**Results:**

There were no significant differences between the five study groups for any of the traits studied (“Somatic Trait Anxiety”, “Psychic Trait Anxiety”, “Stress Susceptibility”, “Detachment” and “Social Desirability”) at any of the occasions. However, the *massage group* showed a significant decrease in the subscale “Somatic Trait Anxiety” (*p* = 0.032), during the entire study period. Significant decreases in the same subscale were also observed in the *pause group* between start and week eight (*p* = 0.040) as well as between week four and week eight (*p* = 0.049) and also in the *control group* between the second and third data collection (*p* = 0.014). The *massage and mental training* group showed a significant decrease in “Stress Susceptibility” between week four and week eight (*p* = 0.022). The *pause group* showed a significant increase in the subscale “Detachment” (*p* = 0.044).

**Conclusions:**

There were no significant differences between the five study groups for any of the traits studied. However, when looking at each individual group separately, positive effects in their levels of “Anxiety”, “Stress Susceptibility” and “Detachment” could be seen. Although the results from this pilot study indicate some positive effects, mechanical chair massage and mental training programmes used in order to increase employee’s ability to recover, needs to be evaluated further as tools to increase the employees ability to recover.

**Australian New Zealand clinical trials registry:**

ACTRN12615000020583, Date of registration: 15/01/2015.

## Background

In many Western countries physical health is in many ways excellent, and people have a long life expectancy. However, the same cannot be said for psychosocial health. Stress-related illnesses, such as burn-out, sleep problems, tension, anxiety and sensitivity to infections or simply a lack of wellbeing have become an increasing problem in recent years. These types of illnesses, such as cardiovascular disorders, type 2 diabetes, reduced immune function and cognitive impairment, typically develop over a very long time and cause much suffering for the affected individuals and can result in long periods of inability to work and in extended sick leave [1, 2]. In Sweden, the most common reason for sick leave is stress. In 2012 15 000 employees were unable to work due to stress-related disorders. In 2014 this number had increased to 26000 employees [3].

A decrease in working people’s ability to recover has among other things been proposed as a key factor in the increasing levels of stress-related health problems in industrialised countries. In fact research has shown that humans can work very hard without any negative effects if they have enough time to recover. On the other hand, if people lose or decrease their ability to recover, they can become more sensitive to work related stress. They can develop chronic fatigue syndrome or even become burned-out with a lower level of work [1, 2, 4–6].

People use different health related activities in order to increase their ability to recover and to lower their stress levels. Two frequently used activities performed by individuals are massage and mental training. Soft massage, i.e., stimulation of sensory nerves in the skin, have been shown to induce anti-stress effects such as reduced anxiety and depression [7, 8]. Back massage applied by an automated massage chair has been shown to produce a general muscle relaxation. This type of artificial massage seems to be especially useful for people who dislike being touched by other people [9].

Techniques for active mental relaxation can make it possible for a person to achieve a relaxed mental state, a state that seems to be linked to reduction of stress and tension and increased potential for recovery and health. Mental relaxation techniques give rise to effects (patterns) that are similar to those induced by massage. In addition, mental training has also been shown to increase creativity and problem solving in relation to negative stress situations [4–6].

According to the World Health Organisation, health promotion is the process of enabling people to increase control over–and improve - their health. Health promotion is not just the responsibility of the health sector but employers also have responsibility for their employees [10]. An increased number of employers have started to work with methods to help their employees stay healthy and be less stressed. One such method is an armchair provided with massage capabilities and mental relaxation programmes. This has been used on more than 7,000 workplaces, both the public and private sector, with 30,000 to 35,000 people using the armchair daily [11]. However, the effects of this armchair have not yet been scientifically evaluated. Therefore evidence is needed to understand the possible effects on health and the underlying mechanism generating these effects. This is important since there is an increasing need for evidence-based health promotion interventions designed to prevent stress-related illness in workplaces [12].

The aim of this study was therefore to evaluate possible effects on employees’ experience of levels of “Anxiety”, “Stress Susceptibility”, “Detachment” and “Social Desirability” when using mechanical massage and mental training programmes, both separately and in combination, during working hours.

## Method

### Study design and setting

The study was performed in the south-west part of Sweden during 2013. In order to get a variation of workplaces, four different workplaces were strategically selected based on their geographical location and working areas, i.e., both small and big towns with employees living in both urban and rural districts and workplaces in both the private and public sector, such as the school and health-care sector as well as the automotive and construction industry.

Randomization occurred at each workplace where each participant was randomly assigned to one of the following five study groups: *i) Massage and mental training* (sitting in the armchair and receiving mechanical massage while listening to the mental training programmes), *ii*) *Massage* (sitting in the armchair and receiving mechanical massage only), *iii*) *Mental training* (sitting in the armchair and listening to the mental training programmes only), *iv*) *Paus* (sitting in the armchair but not receiving mechanical massage or listening to the mental training programmes), *v*). *Control* (not sitting in the armchair at all).

**Fig. 1:**
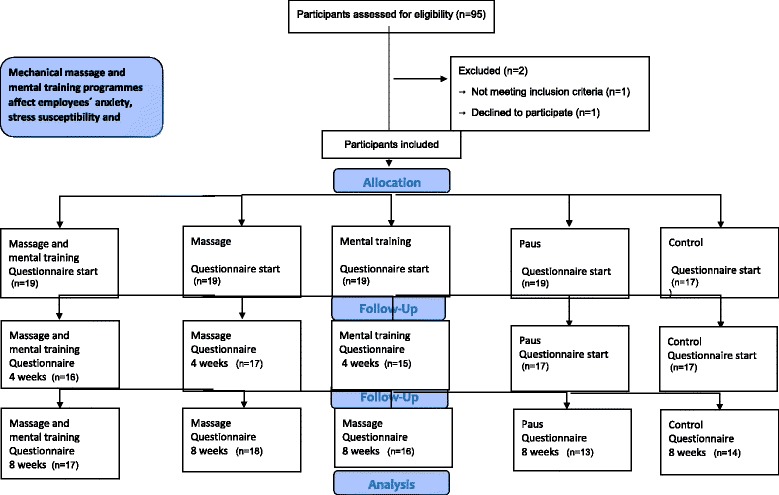
Flow figure of the 93 employees’ participants enrolled in the study, randomly assigned to one of the five study groups

The study lasted for a total of eight weeks. During these weeks the participants in groups *i-iii* took a break from their regular work and sat in the armchair for 15 minutes three times each week, preferably between 1 pm and 4 pm. The study design follows the Consort recommendations (Figure 1) and it is registered in Australian New Zealand Clinical Trials Registry http://www.anzctr.org.au/; ACTRN12615000020583, Date of registration: 15/01/2015.

### Participants

#### Information about the study given to the employers and employees

The selected workplaces was contacted and informed about the study's purpose and meaning. The employees were given the opportunity to ask questions about the study and their possible participation. All employees who chose to participate in the study signed written consent.

#### Inclusion and exclusion criteria

##### Workplaces

Only companies who no prior experience of the armchair was included in the study.

##### Participants

Healthy employees (i.e., without self-reported serious and/or chronic (physical or mental) illnesses and able to perform their work assignment) with no previous experience of using mechanical chair massage and/or the mental training programmes were asked if they wanted to participate in the study. The participants should work between 75 % and 100 % within their own organisations, and they should had a variety of positions and responsibilities. Employees who were pregnant, or who were–at the time–suffering from influenza, colds, fevers or had a skin or kidney disease were excluded from the study due to health risks.

### Intervention

The participants who received mechanical massage (i.e., The *Massage and mental training* group and *Massage* group) all used the same massage programme, but were able to make individual adjustments regarding the strength of the massage. The participants who listened to the mental training programmes (i.e., the *Massage and mental training* group and the *Mental training* group) listened to different programmes in the following order: ”Recovery”–week one, “Mindfulness–learn to live in the present”–week two, ”The way to a better and deeper sleep”–week three, “Reduce the negative stress”–week four, "Learn to think positively”–week five, "Increase your mental strength”–week six, “How to get a greater enjoyment of life”–week seven and ”Recovery”–week eight. The participants in the *Pause* group took a break from their regular work and sat in the chair for 15 minutes three times each week, however they did not use either the massage programme or listen to the mental training programmes. The *Control* group continued their work as usual. In one of the workplaces, due to a hectic schedule, the participants were assigned specific times to use the chair.

### The mechanical massage and mental training programmes

The armchair used in the present study was the “Recovery Chair” included in the “Concept of Recovery” ™, provided by Promas AB, Sweden. The armchair is equipped with the ability to give massages to the neck, shoulders, back and calves. While the user gets a massage he/she may simultaneously listen to a mental training programme (developed and produced by an experienced researcher within mental training, Lars-Eric Uneståhl at Scandinavian International University). The mental training programmes include soft music combined with verbal instructions which are designed to help achieve a relaxing mental state. It is also possible to receive mechanical massage or listen to the mental training programmes separately. All the armchairs were located in a room where a door could be shut, so that that user could be completely separated from other activities while sitting in the chair.

### Outcomes of the study

#### Data collection

On three different occasions during the study period the participants answered a web-based questionnaire (Eva Sys, Alcom System AB, Stockholm, Sweden) regarding their self-experienced physical, mental and social health. The first occasion was at the start of the study, immediately before the randomisation, so that the participants were not influenced by knowing which group they would belong to. The second time was after four weeks and the third time was after eight weeks (end-of-study). At all three times the participants answered the questionnaire after an individual meeting with the researchers at the workplace during regular working hours. When answering the questionnaire the participants were alone in a secluded room.

#### The questionnaire

The statements included in the questionnaire are part of the larger questionnaire, the “Swedish Scale of Personality” (SSP). SSP is a revised form of the previous “Karolinska Scale of Personality” (KSP). In this updated form the scales have been shortened, modernised and psychometrically evaluated. The SSP has been evaluated for more than 3,000 individuals including more than 1,000 patients. SSP contains 13 subscales where each subscale has seven statements with a four-point response format, ranging from “*does not apply at all*” to “*applies completely*” [13]. The following five subscales, and corresponding items, were included in the present study: “Somatic Trait Anxiety” (includes somatic related anxiety such as sweating, teeth clenching or not getting enough air to breathe), “Psychic Trait Anxiety” (includes psychic related anxiety such as an expression of not having much self-confidence, to be worried about things or to be a person who is excessively sensitive and easily hurt), “Stress Susceptibility” (includes aspects of having less energy or feeling more hurried and stressed than most other people, feeling pressure when told to speed up work or having less energy than most other people), “Detachment” (contains social dimensions such as keeping people at a certain distance or being reserved and cold rather than warm and kind) and “Social Desirability” (contains claims such as always being polite and self-controlled, always being a good listener or being willing to admit a mistake).

### Ethical considerations

The study was approved by the Ethics Committee at University of Gothenburg, Sweden (980–12). The Helsinki Declaration was followed [14]. At the staff meeting the research team handed out written information about the project. The employees were informed that they could end the study at any time without giving any reason and that all collected data would only be available to the researchers, not their employer. They were also informed that their workload would not be affected by their participation. The control group were not allowed to use the armchair during the study period. However, once the study had ended they had the opportunity to use the same armchair with the massage and/or mental training programmes for eight weeks.

### Statistical methods

Statistical calculations were performed by using the Statistical Package for the Social Sciences (SPSS, version 22.0). Baseline data is presented as mean and standard deviation (SD) in order to compare similar studies. Since the number of participants in each group was relatively small and a normal distribution could not be taken for granted, non-parametric statistical tests were used to analyse the results. To test for differences between groups on the three separate occasions (start, four weeks and eight weeks) the Kruskal Wallis Test for independent samples, as well as the Mann–Whitney Test for independent samples, were used. To test for differences within each group during the entire study period Friedman’s Two-way Analysis of Variance by Rank was used. When significant, or tendencies to significant, changes were observed with this test the Wilcoxon Signed-Rank Test was used to test for differences between two occasions within the study group. Only answers from individuals who completed the questionnaire at all three time points were included in the analysis. Changes between start and four weeks, between four and eight weeks, as well as between start and eight weeks were analysed. P-values ≤ 0.05 were considered significant and P-values ≤ 0.1 were interpreted as tendencies [15]. Calculation of estimated number of participants was performed based on the results of previous studies using KSP [16, 17].

## Results

A CONSORT flow chart of participant recruitment is shown in Figure 1. Baseline data for the participants is shown in Table 1. Baseline data and the external dropout did not differ significantly between the five groups (data not shown).

**Table 1:** Baseline data for the randomized groups (*n* = 93)

Groups	Massage and mental training	Massage	Mental training	Pause	Control
	(*n* = 19)	(*n* = 19)	(*n* = 19)	(*n* = 19)	(*n* = 17)
Age					
Mean (SD)	50.4 (8.37)	46.5 (12.1)	49.3 (14.1)	47.9 (9.24)	46.6 (10.5)
Sex					
Woman. *n* (%)	16 (84.2)	15 (78.9)	13 (68.4)	13 68.4)	12 (70.6)
Men. *n* (%)	3 (15.8)	4 (21.1)	6 (31.6)	6 (31.6)	5 (29.4)
Marital status					
Single. *n* (%)	3 (16.7)	3 (15.8)	2 (10.5)	2 (10.5)	2 (11.8)
Partner/married. *n* (%)	15 (83.3)	16 (84.2)	17 (89.5)	17 (89.5)	14 (82.4)
Living apart/other. *n* (%)	0	0	0	0	1 (5.9)
Education					
Compulsory School. *n* (%)	1 (5.3)	1 (5.3)	1 (5.3)	0	1 (5.9)
Senior high school. *n* (%)	5 (26.3)	3 (15.8)	2 (10.5)	4 (21.1)	2 (11.8)
	2 (0.5)	3 (15.8)	2 (10.5)	3 (15.8)	1 (5.9)
Higher education. *n* (%)					
University n (%)	11 (57.9)	12 (63.29	14 (73.7)	12 (63.2)	13 (76.5)

### Comparison between the five study groups

There were no significant differences between the five study groups for any of the traits studied (“Somatic Trait Anxiety”, “Psychic Trait Anxiety”, “Stress Susceptibility”, “Detachment” and “Social Desirability”) at any of the occasions.

### Changes within groups

Each group’s mean values with corresponding standard deviations for the subscale studied are presented in Table 2.

**Table 2:** The participant’s answers (mean values (m) and corresponding standard deviations (SD) to the Swedish Scale of Personality (SSP) subscales

Groups	Massage and			Massage			Mental training			Pause			Control		
	Mental training														
	(*n* = 19)			(*n* = 19)			(*n* = 17)			(*n* = 18)			(*n* = 17)		
Week	Start	4	8	Start	4	8	Start	4	8	Start	4	8	Start	4	8
	*n* = 19	*n* = 16	*n* = 18	*n* = 19	*n* = 17	*n* = 18	*n* = 17	*n* = 15	*n* = 16	*n* = 18	*n* = 17	*n* = 13	*n* = 17	*n* = 17	*n* = 14
STA m	1.79	1.79	1.74	2.12^a^	2.01^a,d^	1.92^a,d^	1.85 ^b^	1.88 ^b^	1.68^b,e^	1.80	1.85	1.72	1.71^b^	1.76 ^b^	1.61^b,c^
(SD)	(0.6)	(0.7)	(0.6)	(0.7)		(0.7)				(0.6)	(0.8)	(0.6)		(0.5)	
					(0.7)		(0.6)	(0.5)	(0.5)				(0.5)		(0.5)
PSTA m	2.01	2.06	2.01	1.88	1.91	1.90	1.82	1.82	1.64	1.84	1.90	1.78	1.79	1.79	1.76
(SD)	(0.6)	(0.7)	(0.7)	(0.7)	(0.6)	(0.7)	(0.5)	(0.5)	(0.5)	(0.5)	(0.4)	(0.4)	(0.5)	(0.6)	(0.54)
SS m	2.05^b^	2.14^b^	1.93^b,c^	2.23	2.32	2.28	1.97	2.05	1.96	2.15	2.08	2.04	1.88	1.94	1.84
(SD)	(0.5)	(0.7)	(0.5)	(0.6)	(0.7)	(0.7)	(0.5)	(0.3)	(0.5)	(0.5)	(0.6)	(0.4)	(0.5)	(0.5)	(0.5)
SD m	3.16	3.15	3.18	3.13	3.00	3.08	2.92	2.92	2.88	3.10	2.91	2.98	3.04	3.03	2.98
(SD)	(0.4)	(0.4)	(0.4)	(0.3)	(0.3)	(0.4)	(0.5)	(0.4)	(0.3)	(0.3)	(0.4)	(0.3)	(0.4)	(0.3)	(0.4)
D m	1.84	1.91	1.87	1.70	1.79	1.86	1.95	1.95	1.92	1.85	1.97	2.04	1.71	1.78	1.81
(SD)	(0.6)	(0.6)	(0.6)	(0.5)	(0.4)	(0.5)	(0.6)	(0.5)	(0.5)	(0.6)	(0.5)	(0.5)	(0.4)	(0.5)	(0.4)

Swedish Scale of Personality (SSP) subscales: “Somatic Trait Anxiety” (STA),“Psychic Trait Anxiety” (PSTA), “Stress Susceptibility” (SS), “Social desirability” (SD) and “Detachment” (D). Each subscale has seven statements with a four-point response format, ranging from “does not apply at all” to “applies completely”.

^a^ = Significant change over the entire study period

^b^ = Tendency to change over the entire study period

^c^ = Significant change compared to 4 weeks

^d^ = tendency to change when compared to start

^e^ = tendency to change when compared to 4 weeks

### Massage and mental training group

“Stress Susceptibility” tended to decrease in the massage and mental training group during the entire study period (*p* = 0.088). This decrease became significant during the last four weeks of the study (*p* = 0.022). No other differences were seen for this group.

### Massage group

“Somatic Trait Anxiety” decreased significantly in the massage group (*p* = 0.032) during the entire study period. This decrease was also detected when analysing changes between start and week four (*p* = 0.094) and start and week eight (*p* = 0.050).

### Mental training group

“Somatic Trait Anxiety” tended to decrease in the mental training group during the entire study period (*p* = 0.061). This tendency was also detected when analysing changes between week four and eight (*p* = 0.060).

### Pause group

No changes for any of the traits studied was observed.

### Control group

For the control group there was a tendency to decrease in “Somatic Trait Anxiety” during the entire study period (*p* = 0.076). This decrease reached significance during the last four weeks of the study (*p* = 0.014).

## Discussion

When comparing the different randomised study groups, there were no significant differences between the groups at any of the three time points during the study period. However, the results show several important changes within the separate groups regarding the employee’s experience of “Anxiety”, “Stress Susceptibility” and “Detachment” but not in “Social Desirability” when using mechanical chair massage and mental training programmes, both separately and in combination, during working hours. Although the results from this pilot study indicate some positive effects, mechanical chair massage and mental training programmes used in order to increase employee’s ability to recover, needs to be evaluated further as tools to increase the employees ability to recover.

It appears as if combining mechanical massage with mental training has a reducing effect on employees’ “Stress Susceptibility”. “Stress Susceptibility” includes the aspects of having less energy or feeling more hurried and stressed than most other people. However, since the effect was not generated until the last four weeks of the study a longer period than eight weeks might be needed to generate more sustained effects when both activities are used in combination.

The employees who only received mechanical massage only had a significant decrease in their “Somatic Trait Anxiety” during the entire study period. Thus, a significant reduction in symptoms linked to somatic related anxiety–such as stiffness, tension, teeth clenching and sweating could be seen for the employees who used only the mechanical massage for eight weeks. The results from the present study with mechanical massage coincide with previous studies on traditional massage, which has been shown to have a positive influence on physical health, mental relaxation and sleeping difficulties [18] and increased perception of wellbeing and a decreased perception of pain [19, 20]. The fact that the results from the present study using mechanical massage are similar to those reported for traditional massage indicate that mechanical massage also can be an effective tool for health promotion, especially with regard to experienced somatic health.

The mental training group tended to decrease their anxiety, both somatic and psychic. Previous studies have shown that techniques for mental relaxation can help a person to achieve a relaxed mental state i.e. a reduction of stress and tension and an increased potential for recovery and health [4–6]. The participants in this study changed training programme each week during the study period. How this affected the results can only be speculated upon, but it might be that the changes caused some confusion and the results might have been stronger if the same training programme had been used throughout the entire study.

The pause group, also had a significant decrease in their “Somatic Trait Anxiety”. It has previously been shown that a brief nap taken after lunchtime can positively affect wakefulness, performance, learning ability and furthermore enhances subsequent alertness [21–23]. The result from this study shows that somatic health can also be improved by short breaks during working hours. The pause group also had a significant increase in “Detachment” during the whole study period–i.e. they became less social and less inclined to help others.

In the present study the control group continued with their work as usual, however they still displayed several positive changes during the study period. A possible explanation might be the “Hawthorne Effect”, i.e., the tendency of some people to work harder and perform better when they are participants in an experiment [24]. It is known that positive social interactions can be related to health-promoting effects [25] and this can also be the case in this study, where a large part of the entire workforce belonged to the treatment groups that experienced less stress and/or anxiety.

It also seems that there is a time difference for different techniques to generate effects on different traits. It appears as if the single treatments alone, i.e., mechanical massage only or mental training only, generate effects faster than the combination of the two–as shown by the fact that the effects in the massage group and mental training group were generated throughout the entire study period, whereas the effects in the massage and mental training group appeared first in the second part of the study. This difference in time might be due to people being more used to receiving massage or verbal instructions only and therefore reacting more immediately to these activities, compared to receiving them both simultaneously. Therefore, the first four weeks can perhaps serve as a “break-in period” for the employees who use the combination.

This study included four different types of workplace, since the purpose was to reach a variety of workplaces and workloads. Thus, the generalisation to other workplaces can be limited. In total there were 93 participants that were randomly assigned to one of five different study groups (including a control group). Even if the study population was relatively small–between 17 and 19 participants in each group at occasion I with a reduction at occasions II and III–the randomisation can be regarded as a methodological strength. The study was probably underpowered to detect significant between-group differences, therefore, the results from this explorative pilot study should be seen as a first step towards a larger randomized study within the study area. Due to that this study was a part of a larger study, the appropriate sample size for an adequately powered study should be 107 individuals in each group, in order to detect a 30 % reduction of the individual's cortisol levels in the intervention groups compared with the controls (β = 0.8 and α 0.05) (based on results of this pilot study).

The armchair used in the present study was equipped with the ability to give mechanical massage and playing mental training programmes. Employees who used these functions, either combined or separately, showed improvement in their reported somatic and psychic health. However, further research is needed to investigate the long-term effects of using the armchair and also to investigate its effect on physiological parameters.

### Implications for further studies

Since stress-related illnesses or simply a lack of wellbeing have become an increasing problem there is a need to develop evidence-based treatments for recovery and health promotion in workplaces. Due to the complex nature of stress-related illness, more techniques are needed which can address both somatic and psychic parameters of health at the same time. Based on this study results it can be useful to extend the study period. This is also the recommendation from Back and colleagues who highlighted that the number of weeks of intervention can influence the outcome of studies with massage interventions [26]. It also important to increase the number of participants in each study group and the result from the present study can serve as a base when performing power calculations for future studies.

## Conclusions

There were no significant differences between the five study groups for any of the traits studied (“Anxiety”, “Stress Susceptibility”, “Detachment” and “Social Desirability”). However, when looking at each individual group separately, positive effects in their levels of “Anxiety”, “Stress Susceptibility” and “Detachment” could be seen. Although the results from this pilot study indicate some positive effects, mechanical chair massage and mental training programmes used in order to increase employee’s ability to recover, needs to be evaluated further as tools to increase the employees ability to recover.
